# Factors associated with the health-related quality of life among people with Duchenne muscular dystrophy: a study using the Health Utilities Index (HUI)

**DOI:** 10.1186/s12955-022-02001-0

**Published:** 2022-06-11

**Authors:** Shelagh M. Szabo, Ivana F. Audhya, Basia Rogula, David Feeny, Katherine L. Gooch

**Affiliations:** 1 Broadstreet HEOR, 201 – 343 Railway St, Vancouver, BC Canada; 2 Sarepta Therapeutics, 215 First St, Cambridge, MA 02142 USA; 3 McMaster University and Health Utilities Inc., Hamilton, ON Canada

**Keywords:** Utility, Duchenne muscular dystrophy, DMD, Longitudinal, HUI

## Abstract

**Background:**

Data on health state utility in Duchenne muscular dystrophy (DMD) are few. This study estimated mean utility values by age, ambulatory status and over time, and investigated which aspects of health-related quality-of-life (HRQoL) are most strongly associated with utility in DMD.

**Methods:**

Data from placebo-treated ambulant boys with DMD with exon 51 skip amenable mutations, (NCT01254019), were included. Ambulatory function assessments were conducted at baseline and every 12 weeks for the trial duration. Family member proxies completed the Health Utility Index (HUI) at baseline, 24 and 48 weeks; and HUI3 and HUI2 utility values were summarized. Changes in HUI attribute level over time, and predictors of changes in utility, were explored.

**Results:**

Sixty-one boys (mean [range] age of 8.0 [5–16] years) were included in the analysis. Mean baseline utilities were 0.82 (HUI3) and 0.87 (HUI2); and utilities were 0.35 (HUI3) and 0.55 (HUI2) after loss of ambulation (LOA, where applicable). Over the follow-up period mean utility declined more among the older versus younger boys. Pain accounted for the highest proportion of variability (42%) in change in HUI3 utility from baseline to week 48, while for HUI2, self-care (39%) did. After LOA, change in ambulation levels explained 88% of the decline in mean HUI3 utility and change in mobility levels explained 66% of the decline in mean HUI2 utility.

**Conclusions:**

Utility values among this sample were higher than previously published estimates. In younger boys utility remained relatively stable, but older boys and those losing ambulation experienced important declines over follow-up.

**Supplementary Information:**

The online version contains supplementary material available at 10.1186/s12955-022-02001-0.

## Background

Duchenne muscular dystrophy (DMD) is a rare progressive neuromuscular disorder caused by mutations in the gene for dystrophin, a protein required for the structural integrity of muscle cells [1–4]. Affected patients typically present in early childhood with gait abnormalities, muscle weakness, and delayed motor and cognitive function [1, 5–8]. Among those with DMD, progressive muscular weakness leads to loss of ambulation (LOA) and scoliosis in late childhood, and loss of upper limb function in early adulthood. Additionally, loss of strength in active breathing muscles contributes to respiratory insufficiency and the need for ventilation in the teenage years. Cardiomyopathy also develops in late adolescence and the progression of DMD typically culminates with early mortality in the third or fourth decade of life [9].

The impact of DMD on daily life results in those with DMD often being dependent on family caregivers (and sometimes other caregivers) to manage their everyday needs. Given its severity and progressive nature, DMD has a substantial impact on the health-related quality of life (HRQoL) of both patients with DMD, and their caregivers [10]. In general, data on health state utility in DMD are relatively limited with most data derived from preference-based measures such as the EQ-5D and Health Utilities Index (HUI). However, data from assessments using disease-specific instruments (e.g. the DMD-QOL) are now starting to become available [10–12].

The aspects of HRQoL that are affected by DMD and impact patient utility are not well understood [13]. At present, utility data exist for only a few health states, largely defined by ambulatory status, which is a substantial limitation; as is the cross-sectional nature of the studies from which the data are derived. It is also unclear, at present, which aspects of DMD and its progression contribute most to utility among those with DMD. In order to address these gaps, this study sought to estimate mean health state utility values by age, ambulatory status and over time among boys with DMD; and to investigate which HRQoL attributes are most strongly associated with health state utility among patients with DMD.

## Methods

Data derived from ambulant boys with DMD aged 5 years or older, with exon 51 skip amenable mutations, randomized to the placebo arm of the DEMAND trial (NCT01254019; provided by BioMarin Pharmaceuticals Inc), were included. The study inclusion criteria required participants be able to complete the six-minute walk distance (6MWD) of ≥ 75 m at each pre-drug visit [14]. The study was conducted between December 30, 2010 and June 28, 2013, with the follow-up period of 48 weeks from baseline/ randomization; [15].

### Outcome measures

To measure ambulatory function, the North Star Ambulatory Assessment (NSAA), timed rising from floor (RFF), 10-m timed walk/run (10MWR), and 6MWD tests were conducted by trained assessors at baseline and every 12 weeks. The NSAA is a 17-item functional assessment scale (range, 0 to 34) designed for ambulant boys with DMD, where performance on tests related to ambulatory function (e.g. box climbing, lifting head, running) are assessed on a 3 point scale (0 = unable to achieve independently to 2 = "Normal"—no obvious modification of activity) [16]. Total scores are calculated by summing the scores for the individual items. The NSAA takes approximately 10 min to complete [17], and its validity, feasibility and reliability have previously been demonstrated [17–19]. Conducted as either alongside or independent from the NSAA, the timed RFF test measures the time taken to raise from supine to standing and the 10MWR test, the time spent for walking/running 10 m.[16] Definitions and explanations of scoring by measure are presented in Additional file 1: Appendix Table 1. If loss of ambulation (LOA) occurred within the study period, which was classified by trained assessors based on a patient’s inability to perform study ambulatory assessments, the date at which it was observed was recorded.

Family members serving as proxy respondents also completed the 15-item HUI questionnaire, a preference-based utility measure, at screening, baseline, 24 weeks, and 48 weeks (or early withdrawal). The same proxy respondent was asked to complete the HUI at each assessment visit, and the recall period of the questionnaire was the past four weeks. HUI responses are used to quantify health utility (on a scale of 0 [dead] to 1 [full health]) according to two complementary health-status classification systems, the HUI mark 3 (HUI3) and HUI mark 2 (HUI2) [20]. The HUI3 system considers eight attributes: vision, hearing, speech, ambulation, dexterity, emotion, cognition, and pain. The HUI2 system considers seven attributes: sensation (vision, hearing, and speech), mobility, emotion, cognition, selfcare, pain, and fertility. Fertility is optional and was not assessed. Levels of impairment for the eight HUI3 attributes range from 1 (no impairment) to 5 or 6 (severe impairment); and for the six relevant HUI2 attributes, from 1 (no impairment) to 4 or 5 (severe impairment). For both HUI3 and HUI2 attributes, increasing attribute levels therefore indicate worsening function. HUI3 and HUI2 attribute levels are transformed using the developers’ algorithm to generate an overall HUI3 and HUI2 utility score [21].

### Analysis

Baseline characteristics of the sample were summarized. HUI3 and HUI2 attribute levels were calculated for each patient at baseline, 24 weeks, and 48 weeks (where this was possible, given availability of the required responses from the HUI questionnaire at each time point). HUI3 and HUI2 utility values were then estimated for each patient at these same visits following the developers’ algorithm and scoring instruction [21]. To summarize HUI utility at baseline and over time, mean (standard deviation [SD]) utility values were calculated at baseline, 24 weeks, and 48 weeks. Mean utility values and changes in utility values (from baseline to 48 weeks) were compared between younger (5 to < 8 years) and older (8 to 16 years) patients, with age calculated at baseline.

To understand the scores on HUI attribute levels that fed into the HUI utility calculations, mean (SD) HUI attribute levels were computed. The proportion of patients with moderate or severe impairments in each attribute was calculated, using attribute-specific definitions provided by the developers [21] and patient counts were tabulated by the number of attributes affected (these specific calculations were performed using baseline measures only).

To investigate variability in changes in HUI utility from baseline to 48 weeks, the percent of variation around mean changes in HUI3 and HUI2 utility explained by changes in each attribute was estimated using the R^2^ measure from linear regression analyses, with a single attribute as a predictor variable. To find the percent of variation explained by a combination of attributes, all the attributes were entered into a single linear regression analysis as predictors. To understand how HUI attribute levels changed over time, the proportions of patients with worsening, no change, and improvement in level of each HUI3 and HUI2 attribute at 48 weeks vs baseline were calculated. The attributes with the largest proportion of patients with worsening and with improvement were determined.

To understand which attributes were driving large changes in utility (i.e. change in utility of 0.2), the proportions of patients with worsening and improvement in each HUI3 and HUI2 attribute were calculated separately among patients with large declines or improvements in utility. Patients with a utility change of at least 0.2 were considered in a primary analysis, and patients with a change of at least 0.1 and 0.03 (considered a clinically meaningful change in utility on the HUI measure) [20] were considered as additional sensitivity analyses. Among the patients experiencing declines in utility, the attributes with the largest proportion of patients with worsening levels were determined. Similarly, among patients experiencing improvements in utility, the attributes with the largest proportion of patients with improvement in levels were determined. For patients with large declines or improvements in utility, mean (SD) utility at baseline was calculated.

Some patients lost ambulation during the course of the trial and the number of patients in whom this occurred over the 48-week period was determined. Among patients with LOA, mean (SD) utility values were calculated at baseline and post-LOA. Estimates post-LOA were calculated by first finding means at the patient-level across visits, then finding the mean (SD) across patients. The mean change in utility from baseline to after LOA was also calculated and a smoothing line was fitted to utility values, with time relative to LOA on the x-axis. The percent of the decline in mean utility after LOA explained by mean change in each attribute level was determined by direct calculation via the HUI scoring algorithm.

## Results

### Patient characteristics

A total of 61 ambulant boys with DMD were included in this analysis. At baseline, the mean (range) age was 8.0 (5–16) years (Table 1), the mean (SD) NSAA score was 21 (8) and ranged from 4 to 33, the mean (SD) 6MWD was 348 m and ranged from 108 to 566 m and the mean (SD) timed 10MWR test was 7.5 s and ranged from 3 to 20 s. Nineteen (31%) patients were unable to RFF independently, at baseline, six (10%) of patients were unable to RFF even with use of a chair. Among patients with the ability to RFF (including those who could not RFF without a chair; n = 55), mean timed RFF was 13 s.

**Table 1:** Baseline characteristics

	DMD sample (n = 61)
Age, years	
Mean (SD)	8.0 (2.4)
Median (IQR)	8 (6, 9)
Min, max	5, 16
By group, n (%)*	
5–7 years	29 (48)
8–11 years	29 (48)
12–16 years	3 (5)
NSAA total score	
Mean (SD)	21.0 (8.1)
Median (IQR)	23 (15, 27)
Min, max	4, 33
6MWD (m)	
Mean (SD)	348 (92)
Median (IQR)	354 (311, 400)
Min, max	108, 566
Timed 10MWR test (s)†	
Mean (SD)	7.5 (3.6)
Median (IQR)	6.1 (5.2, 8.5)
Min, max	3.4, 20.0
Timed RFF (s)†	
Mean (SD)	13.4 (15.9)
Median (IQR)	6.8 (4.2, 12.0)
Min, max	0.5, 63.0
RFF	
Unable to RFF independently, n (%)	19 (31)
Unable to RFF, even with use of a chair, n (%)	6 (10)

6MWD = six-minute walk distance; NSAA = North Star Ambulatory Assessment; RFF = rising from floor; SD = standard deviation

*While age categories 8-11y and 12-16y were initially investigated, the three patients in the 12-16y category were considered along with the 8-11y old boys, due to small sample size

†n = 55

### Health state utility

**Fig. 1:**
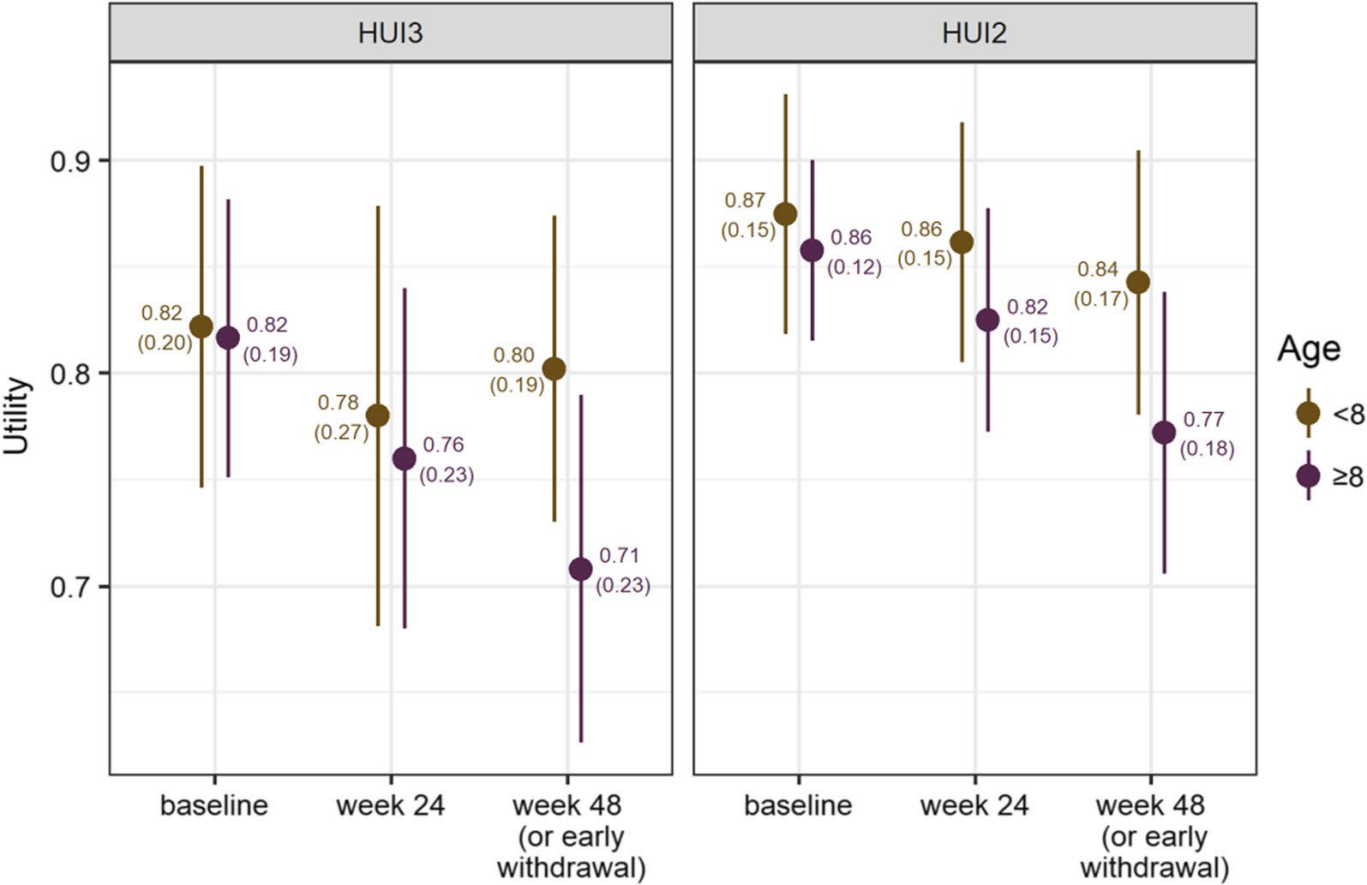
Mean (SD) HUI3 (left) and HUI2 (right) utility by visit, stratified by baseline age. Whiskers represent 95% confidence intervals

At baseline, mean (SD) HUI3 utility was 0.82 (0.19) and HUI2 utility was 0.87 (0.13) in the overall group. Mean (SD) baseline utilities were similar between younger and older boys (Fig. 1). The mean (SD) HUI3 utility value was 0.82 (0.20) among boys aged 5 to < 8 years (n = 28), and 0.82 (0.19) among boys 8 to 16 years (n = 32). The mean (SD) HUI2 utility value 0.87 (0.15) among boys aged 5 to < 8 years (n = 27), and 0.86 (0.12) those 8 to 16 years (n = 31). At 48 weeks, mean (SD) HUI3 utility was 0.75 (0.22) and HUI2 utility was 0.81 (0.18). Mean (SD) utility change at 48 weeks in the overall group was -0.06 (0.19) for HUI3 and -0.05 (0.14) for HUI2, with mean utility having declined more among the older boys than the younger boys (Fig. 1). HUI3 utility was 0.80 (0.19) among those 5 to < 8 (n = 28) compared with 0.71 (0.23) among those aged 8 to 16 years (n = 30) and for HUI2, 0.84 (0.17) among those 5 to < 8 (n = 28), compared to 0.77 (0.18) among those aged 8 to 16 years (n = 29).

### HUI attribute levels

Mean (SD) HUI3 attribute levels at baseline were: vision, 1.03 (0.18); hearing, 1.00 (0.00); speech, 1.08 (0.28); ambulation, 1.68 (0.91); dexterity, 1.22 (0.76); emotion, 1.12 (0.32); cognition, 1.37 (0.94); and pain, 1.77 (0.72). Of the HUI3 attributes, moderate or severe impairments at baseline were reported more frequently for attributes such as pain (in 17% of patients), ambulation (13%), and cognition (7%), compared to vision, hearing or speech for example (all 0%;Table 2). Twenty-three percent of patients had at least some impairment in ≥ 3 attributes at baseline; 57% had at least some impairment in 1 or 2 attributes; and 20% had no evidence of impairment, across all attributes (Additional file 2: Appendix Table 2).

**Table 2:** HUI3 and HUI2 attribute levels at baseline (n = 60)*

HUI3 attributes, n(%)								
Levels	Vision	Hearing	Speech	Ambulation	Dexterity	Emotion	Cognition	Pain
1 (normal)	58 (96.7)	60 (100.0)	55 (91.7)	32 (53.3)	54 (90.0)	53 (88.3)	48 (80.0)	24 (40.0)
2	2 (3.3)	0 (0.0)	5 (8.3)	20 (33.3)	3 (5.0)	7 (11.7)	8 (13.3)	26 (43.3)
3	0 (0.0)	0 (0.0)	0 (0.0)	3 (5.0)	0 (0.0)	0 (0.0)	0 (0.0)	10 (16.7)
4	0 (0.0)	0 (0.0)	0 (0.0)	5 (8.3)	2 (3.3)	0 (0.0)	3 (5.0)	0 (0.0)
5	0 (0.0)	0 (0.0)	0 (0.0)	0 (0.0)	1 (1.7)	0 (0.0)	0 (0.0)	0 (0.0)
6	0 (0.0)	0 (0.0)	-	0 (0.0)	0 (0.0)	-	1 (1.7)	-
Impairment category								
None or mild	60 (100.0)	60 (100.0)	60 (100.0)	52 (86.7)	57 (95.0)	60 (100.0)	56 (93.3)	50 (83.3)
Moderate or severe	0 (0.0)	0 (0.0)	0 (0.0)	8 (13.3)	3 (5.0)	0 (0.0)	4 (6.7)	10 (16.7)

HUI2 attributes, n(%)						
Levels	Sensation	Mobility	Emotion	Cognition	Self-care†	Pain†
1 (normal)	53 (88.3)	32 (53.3)	39 (65.0)	48 (80.0)	34 (57.6)	33 (55.9)
2	2 (3.3)	20 (33.3)	18 (30.0)	11 (18.3)	16 (27.1)	25 (42.4)
3	5 (8.3)	8 (13.3)	2 (3.3)	0 (0.0)	2 (3.4)	1 (1.7)
4	0 (0.0)	0 (0.0)	0 (0.0)	1 (1.7)	7 (11.9)	0 (0.0)
5	-	0 (0.0)	1 (1.7)	-	-	0 (0.0)
Impairment category						
None or mild	55 (91.7)	52 (86.7)	57 (95.0)	59 (98.3)	50 (84.7)	58 (98.3)
Moderate or severe	5 (8.3)	8 (13.3)	3 (5.0)	1 (1.7)	9 (15.3)	1 (1.7)

*1 patient was missing all HUI3 and HUI2 information at baseline. For HUI2, the †self-care and pain attributes each had an additional patient missing a value

NOTE for all HUI2 and HUI3 attributes, increasing values indicate worsening levels

Mean (SD) HUI2 attribute levels at baseline were: sensation, 1.21 (0.59); mobility, 1.62 (0.72); emotion, 1.36 (0.55); cognition, 1.22 (0.53); self-care, 1.69 (1.01); and pain, 1.45 (0.54). Of the HUI2 attributes, moderate or severe impairments at baseline were reported more frequently for attributes such as self-care (15%), mobility (13%), and sensation (8%) compared to cognition and pain for example (1.7% each; Table 2). At baseline, 32% of patients had at least some impairment in ≥ 3 attributes; 47% had at least some impairment in 1 or 2 attributes; and 21% had no evidence of impairment, across all attributes (Additional file 2: Appendix Table 2).

### Change in HUI attributes over time

**Fig. 2:**
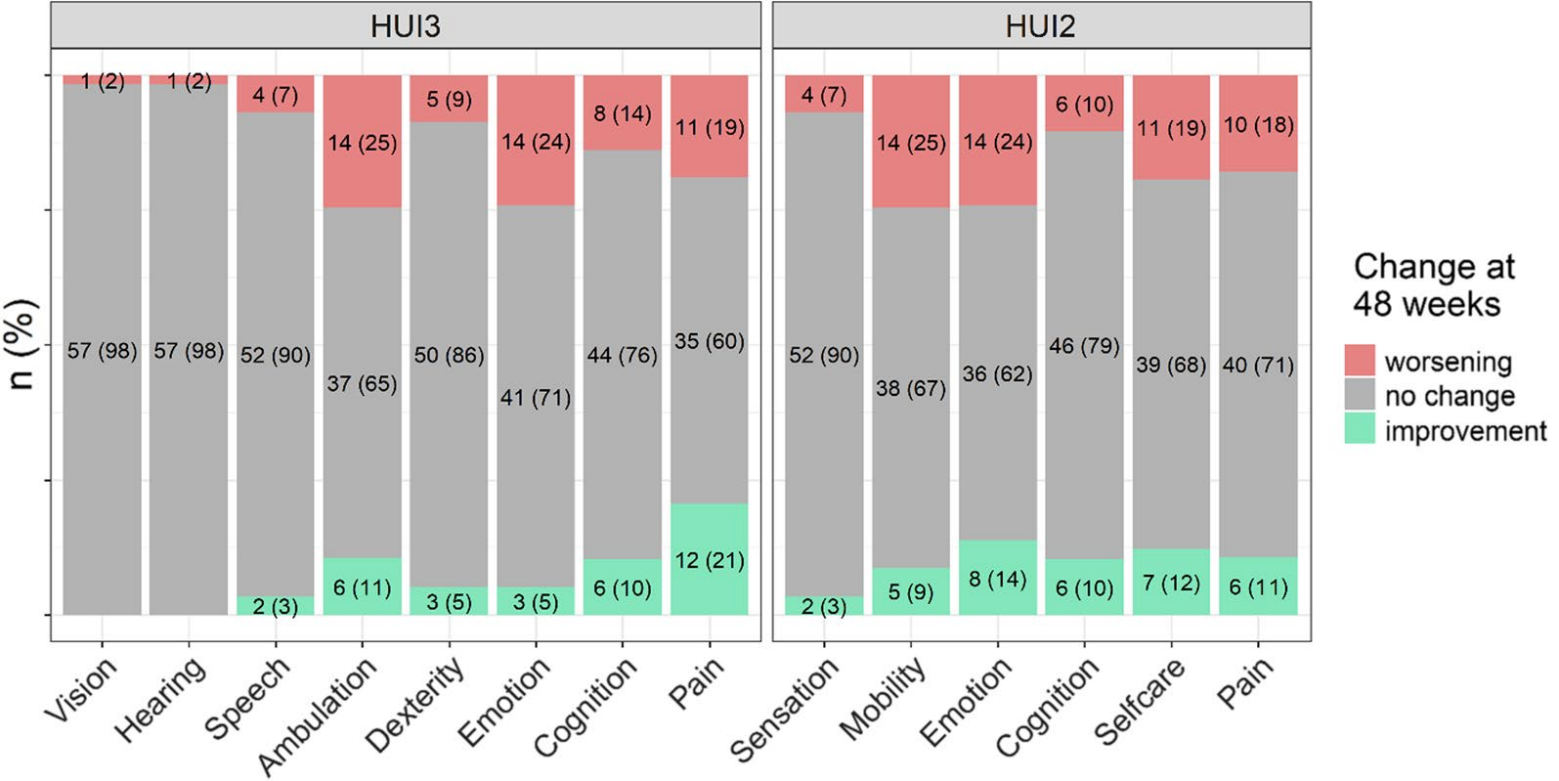
Changes in HUI attribute levels from baseline to week 48 among the total sample of patients

From baseline to 48 weeks, 25% of patients worsened in ambulation, 24% in emotion, 19% in pain, and 14% in cognition on the HUI3. Over the same period, 21% of patients experienced an improvement in pain, 11% in ambulation, and 10% in cognition on the HUI3 (see Fig. 2 for the proportion worsening or improving across the entire set of HUI3 attributes). On the HUI2, 25% of patients worsened in mobility, 24% in emotion, 19% in self-care, and 18% in pain from baseline to 48 weeks. Over the same period, 14% of patients improved in emotion, 12% in self-care, 11% in pain, and 10% in cognition on the HUI2 (see Fig. 2 for the proportion worsening or improving across the entire set of HUI2 attributes).

### Predictors of changes in health state utility

**Fig. 3:**
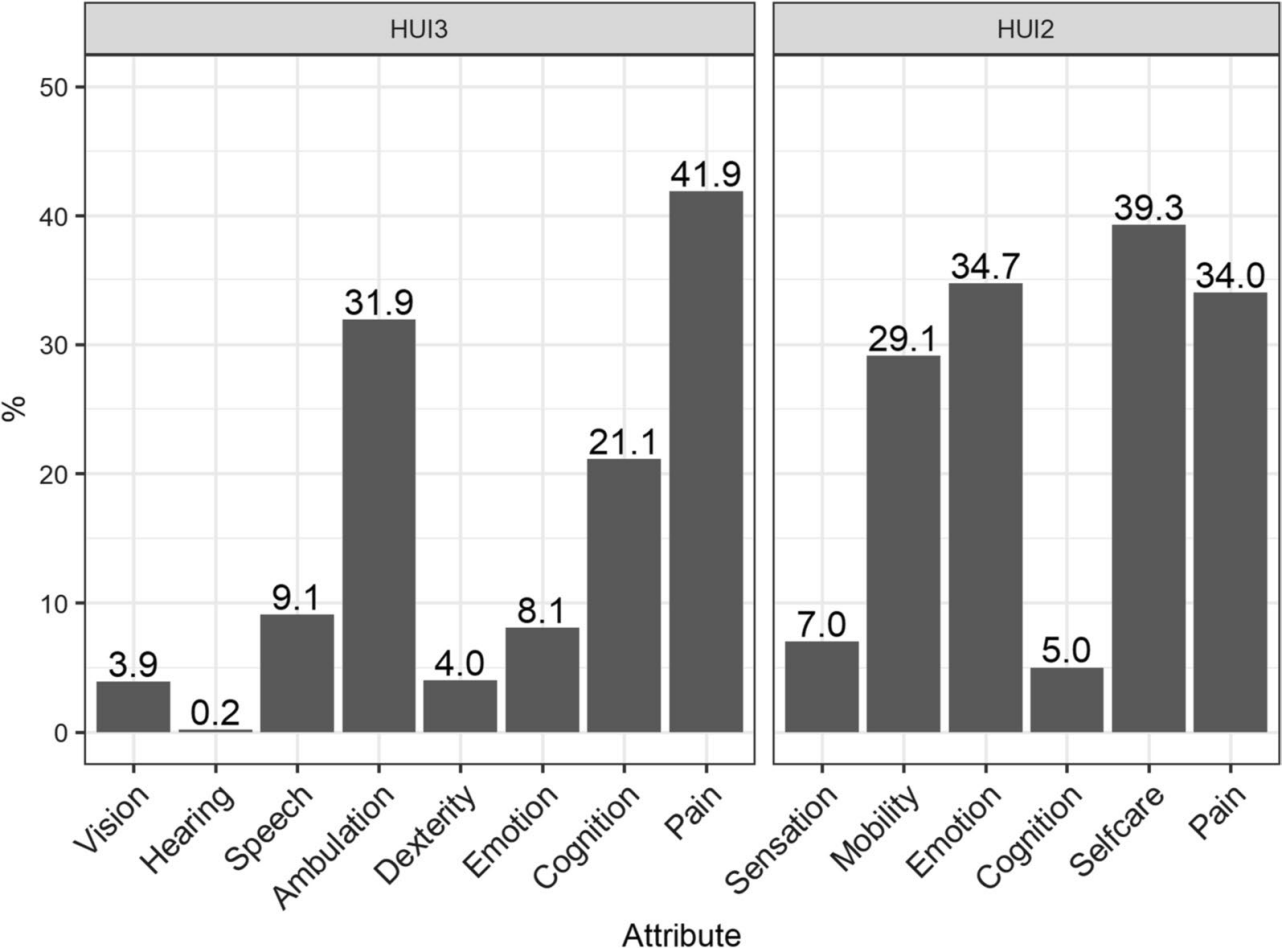
Percent of variability in changes HUI utility, across patients, explained by changes in each attribute

The attributes explaining the largest proportions of variability in changes in overall HUI3 utility scores from baseline to 48 weeks were pain (42%), ambulation (32%), and cognition (21%; Fig. 3). When included together in a single regression model, these attributes explained 67% of variability in changes in utility across the study sample. The HUI2 attributes which explained the largest proportions of variability in changes in HUI2 utility scores from baseline to 48 weeks were self-care (39%), emotion (35%), pain (34% Fig. 3), and mobility (29%). When included together in a single regression model, these attributes explained 85% of variability in changes in utility across the study sample.

**Fig. 4:**
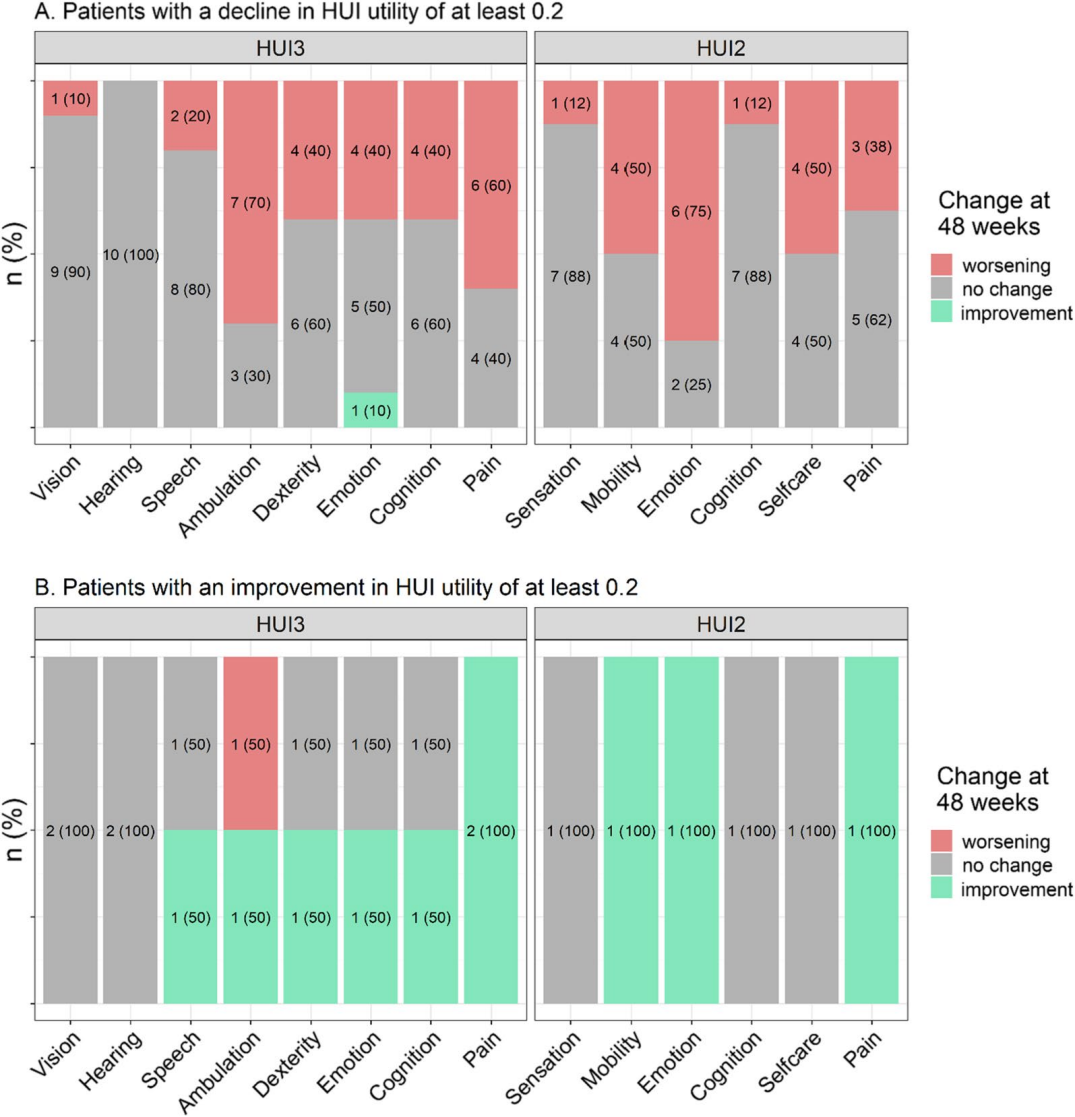
Changes in HUI attribute levels from baseline to week 48 among patients with **a** a decline in HUI utility of at least 0.2, and **b** an improvement in HUI utility of at least 0.2, between baseline and week 4

Among patients with large improvements and declines in utility (at 48 weeks vs. baseline), the proportions of patients with worsening and improvement in levels for each HUI3 and HUI2 attribute were calculated (Fig. 4). Ten patients experienced a decline in HUI3 utility of 0.2 + ; with primary worsening in ambulation (70%), pain (60%), and dexterity/emotion/cognition (all 40%); and with mean (SD) utility at baseline of 0.88 (0.12). Further, three of these ten patients lost ambulation over the 48 weeks. Eight patients experienced a decline in HUI2 utility of 0.2 + ; with the largest proportion experiencing worsening in emotion (75%), mobility (50%), self-care (50%), and pain (38%). Among them, mean (SD) utility at baseline was 0.90 (0.07). Two of these eight patients lost ambulation over the 48 weeks. It is interesting to note that these two patients are among the three who lost ambulation and had a decline in HUI3 utility of 0.2 + . The third patient had a large decline in HUI3 utility (0.62), and a much smaller decline in HUI2 utility (0.09). While this patient had a 2-level worsening in HUI3 emotion, they had a 1-level improvement in HUI2 emotion.

Two patients experienced an improvement in HUI3 utility of 0.2 +; these patients both improved in pain, and one of the two also improved in speech, ambulation, dexterity, emotion, and cognition. One patient experienced an improvement in HUI2 utility of 0.2 +; this patient had improvement in mobility, emotion, and pain. The sensitivity analysis examining patients with declines or improvements in utility of at least 0.1 and 0.03 showed similar trends although the proportions of patients experiencing worsening in attribute levels were generally smaller (Additional file 5: Appendix Fig. 1). Additional file 3: Appendix table 3 and Additional file 4: Appendix table 4 show observed HUI utility changes and corresponding attribute changes at the patient-level.

### Health state utility among those with change in ambulatory status

Six boys (9.8%) lost ambulation over the 48-week period; their mean (SD) utility at baseline was 0.60 (0.39) for HUI3 and 0.69 (0.14) for HUI2. Their mean (SD) utility after LOA was 0.35 (0.11) for HUI3 and 0.55 (0.07) for HUI2. The mean (SD) decline in utility after LOA was 0.25 (0.32) for HUI3 and 0.15 (0.12) for HUI2. There was a suggestion of a plateauing in utility after LOA (Additional file 6: Appendix Fig. 2). Change in ambulation and mobility levels were the largest contributors to declines in utility. Change in ambulation levels explained 88% of the decline in HUI3 utility after LOA, while change in emotional status explained 11%. Change in mobility levels explained 66% of the decline in HUI2 utility after LOA, while change in self-care status explained 34%.

## Discussion

There are few data presently available on utility values relevant to those with DMD. Existing generic preference-based data are cross-sectional and reflect the impact of living in a limited range of health states [13], and DMD-specific measures are under development and not yet widely used [12]. Nonetheless rigorous utility data for health states that represent the breadth of patient experiences are required for assessing the value of new therapies in economic models based on the quality-adjusted life year (QALY) [22, 23]. Published estimates for ambulant boys with DMD, which were all assessed using the HUI3, range from 0.65 to 0.75; and for non-ambulant boys from 0.05 (HUI3) to 0.44 (EQ-5D) [24–26]. While existing utility values document the substantial HRQoL impacts of DMD, longitudinal estimates from a clinically well-characterized sample are lacking; as is an understanding of which factors drive changes in utility in DMD.

Utility scores among this sample of boys with DMD, followed for almost one year, were found to be higher for both ambulant and non-ambulant boys compared to published HUI3 and HUI2 estimates. Differences between these and other published estimates could be due to differences in study populations included and the exact measures employed; and it is important to note that the inclusion criteria for the present study were designed to focus on boys who met some thresholds for ambulatory function at baseline. In the present study, older boys and boys who lost ambulation experienced the largest losses in utility over the 48 week follow up period. However, there was considerable variability in utility values, as well as in the magnitude of change in utility, over time.

The HUI measures document health status using a multi-attribute health status classification system– including aspects of ambulatory, physical, cognitive, and sensory function; social and emotional health; and attributes such as ability to complete self-care activities and presence of pain [20]. This questionnaire therefore provides rich data by which to obtain a coherent view of the relationship between HRQoL and DMD as well as understand predictors of variability in utility. These findings indicate that the attributes of pain, ambulation/mobility, self-care, and emotion were the best predictors of changes in utility in this sample of ambulatory boys with DMD, as they explained the largest proportion of variability in utility over time. The importance of ambulatory function and mobility in explaining utility has clinical face validity, given the neuromuscular impairments are the core symptoms experienced by boys with DMD. It is interesting to note that these tended to be the attributes with the largest proportion of patients with moderate to severe impairments at baseline (except for HUI2 pain and emotion). That is consistent with the finding that attributes such as vision—measured by the HUI3 but not typically affected by progression in DMD—would likely remain steady at high level in patients over the course of the follow up period. Ambulation, cognition, pain, dexterity, self-care, mobility, and notably also emotion, were the attributes with the largest proportions of patients experiencing worsening in their levels over the course of the study. Because of the observed contribution of various attributes, including emotion, pain, ambulation/mobility, and self-care in explaining changes in utility, it is clear that utility declines in DMD are multifactorial and reflect the impact of numerous aspects of patient wellbeing.

The HUI is a group of generic, multi-attribute, and preference-based systems for the purposes of measuring health status. Item responses to the various questions contribute to two distinct utility values (a HUI2 utility or HUI3 utility), and across the two systems, attributes with the same name address different underlying constructs [20]. For example, HUI2 emotion focuses on worry and anxiety while HUI3 emotion focuses on happiness and depression; HUI2 pain considers frequency of pain and type of pain control required, and HUI3 pain considers pain severity and activity disruptions due to pain. Thus, one’s emotional status for example can contribute differently to a HUI2 vs HUI3 utility score because of the different dimensions of emotion considered within the two classification systems [20]. Of particular relevance to DMD is that the HUI2 mobility attribute reflects a combination of HUI3 ambulation and dexterity and thus there is an interdependency between these aspects as they contribute to an individual’s HUI2 and HUI3 utility values. The two HUI systems are independent but complementary. While HUI3 utility values are often reported on their own including in DMD [13], the HUI2 system does offer distinct, independent attributes with high relevance to DMD, including self-care and an emotional attribute focusing on worry and anxiety [20].

Strengths of this study include the large sample of ambulatory boys with this rare disease included, and the longitudinal design. This sample is clinically well-characterized at baseline, on a variety of functional measures with high relevance to DMD. How changes in physical function over time are related to change in utility in DMD represents an important future direction. Limitations to the study include the relatively small sample size in selected subgroups (for example, to understand the utility among those experiencing LOA). Additionally, as all boys included in this study were skip 51 amenable, findings may not be generalizable to boys with other genotypic mutations. Further, the results are based on family members serving as proxy respondents. It is possible that utility as reported by the patients themselves might differ. Similarly rigorous longitudinal data including utility assessments that describe patients in a wider variety of health states in DMD are needed to, for example, understand the impact of varying levels of upper limb function, or respiratory status, on health state utility. Finally, the suitability and comprehensiveness of the generic preference-based measures to assess the HRQoL impact of DMD among those with DMD deserves consideration. A systematic review and other recent investigations conducted after the data collection period of the present study, highlighted the lack of evidence to support structural and content validity of the HUI and other generic instruments for assessing HRQoL impacts among those with DMD. Additionally, how well existing generic instruments cover the aspects of HRQoL most important from the patient perspective, particularly from pediatric populations,is now emerging [11, 27–30]. Additional work to validate the content of existing generic measures and confirm that included constructs have relevance to the DMD population, as well as the development of novel DMD-specific measures [11, 12, 28], will help ensure that HRQoL among those with DMD can be reliably and validly measured. It will also be important to ensure that all relevant aspects of HRQoL that may be affected by DMD progression are being captured.

## Conclusions

Despite variability in scores, utility values among this sample of ambulatory boys with DMD were relatively high, and higher than previously published estimates. While the utility of younger boys remained relatively stable, older boys and those losing ambulation experienced important declines over follow-up. These findings are valuable in augmenting scarce data on health state utilities for ambulatory boys with DMD.

## Supplementary Information

**Additional file 1: Appendix Table 1.** Measure scoring.

**Additional file 2:Appendix Table 2.** Number of HUI3 and HUI2 attributes affected (not level 1) at baseline.

**Additional file 3:** **Appendix Table 3.** Observed changes in HUI3 utility and attribute levels from baseline to week 48, with each row representing an individual patient.

**Additional file 4:** **Appendix Table 4.** Observed changes in HUI2 utility and attribute levels from baseline to week 48, with each row representing an individual patient.

**Additional file 5:** **Appendix Figure 1.** Changes in HUI attribute levels from baseline to week 48 among patients with (A) a decline in HUI utility of at least 0.1, (B) an improvement in HUI utility of at least 0.1, (C) a decline in HUI utility of at least 0.03, and (D) an improvement in HUI utility of at least 0.03, between baseline and week 48.

**Additional file 6.** **Appendix Figure 2.** HUI3 and HUI2 utility by time since LOA, with a black smoothing line and colors denoting individual patients (n=6).

## Data Availability

The dataset supporting the conclusions of this article is included within the article’s additional files.

## Abbreviations

10MWR: 10-meter timed walk/run
6MWD: Six-minute walk distance
DMD: Duchenne muscular dystrophy
HUI: Health Utilities Index
HRQoL: Health-related quality of life
IQR: Interquartile range
LOA: Loss of ambulation
NSAA: North Star Ambulatory Assessment
QALY: Quality-adjusted life year
QOL: Quality of life
RFF: Timed rising from floor
SD: Standard deviation
