# Supplementary material for: Factors associated with the health-related quality of life among people with Duchenne muscular dystrophy: a study using the Health Utilities Index (HUI)
Source: Health Qual Life Outcomes. 2022 Jun 11;20:93. doi: 10.1186/s12955-022-02001-0 (PMC9188127; doi:10.1186/s12955-022-02001-0)
Supplement: Supplementary file 1 — Additional file 1: Appendix Table 1. Measure scoring. [file 12955_2022_2001_MOESM1_ESM.docx]

Appendix table 1: Measure scoring

| **10 meter walk/run (10MWR)** | |
| --- | --- |
| **Grade** | **Description** |
| 1 | Unable to walk independently |
| 2 | Unable to walk independently, but can walk with KAFOs or support from person |
| 3 | Highly adapted wide based lordotic gait. Cannot increase walking speed |
| 4 | Moderately adapted gait. Can pick up speed but cannot run |
| 5 | Able to pick up speed, but runs with double stance phase |
| 6 | Runs and gets off both feet off the ground (with no double stance phase) |
| **Rise from floor (RFF)** | |
| **Grade** | **Description** |
| 1 | Unable to stand from supine, even with use of a chair |
| 2 | Assisted Gowers-requires furniture for help in arising from supine to upright |
| 3 | Full Gowers-rolls over, stands up with both hands "climbing up“ legs to upright |
| 4 | Half Gowers-rolls over, stands up with one hand support on leg |
| 5 | Rolls to side and/or stands up with hands on floor to rise, does not touch legs |
| 6 | Stands up without rolling over |
| **NSAA scores** | |
| NSAA scores range from 0 to 34 and are calculated as a sum of 17 activity grades, each with possible values 0 (Unable to achieve independently), 1 (Modified method but achieves goal independent of physical assistance from another), or 2 (Normal – no obvious modification of activity). | |
