# Supplementary material for: Factors associated with the health-related quality of life among people with Duchenne muscular dystrophy: a study using the Health Utilities Index (HUI)
Source: Health Qual Life Outcomes. 2022 Jun 11;20:93. doi: 10.1186/s12955-022-02001-0 (PMC9188127; doi:10.1186/s12955-022-02001-0)
Supplement: Supplementary file 2 — Additional file 2:Appendix Table 2. Number of HUI3 and HUI2 attributes affected (not level 1) at baseline. [file 12955_2022_2001_MOESM2_ESM.docx]

Appendix table 2: Number of HUI3 and HUI2 attributes affected (not level 1) at baseline

| **Number of attributes affected** | **HUI3 (n=60)*** | **HUI2 (n=58)*** |
| --- | --- | --- |
|  | **n (%)** | **n (%)** |
| 0 | 12 (20.0) | 12 (20.7) |
| 1 | 20 (33.3) | 15 (25.9) |
| 2 | 14 (23.3) | 12 (20.7) |
| 3 | 9 (15.0) | 8 (13.8) |
| 4 | 4 (6.7) | 5 (8.6) |
| 5 | 1 (1.7) | 5 (8.6) |
| 6 | 0 (0.0) | 1 (1.7) |
| 7 | 0 (0.0) |  |
| 8 | 0 (0.0) |  |

* 1 patient had incomplete HUI3 information at baseline and 3 patients had incomplete HUI2 information at baseline.
