# Supplementary material for: Factors associated with the health-related quality of life among people with Duchenne muscular dystrophy: a study using the Health Utilities Index (HUI)
Source: Health Qual Life Outcomes. 2022 Jun 11;20:93. doi: 10.1186/s12955-022-02001-0 (PMC9188127; doi:10.1186/s12955-022-02001-0)
Supplement: Supplementary file 3 — Additional file 3: Appendix Table 3. Observed changes in HUI3 utility and attribute levels from baseline to week 48, with each row representing an individual patient. [file 12955_2022_2001_MOESM3_ESM.docx]

Appendix table 3: Observed changes in HUI3 utility and attribute levels from baseline to week 48, with each row representing an individual patient.

| **HUI3 utility change from baseline to week 48** | **HUI3 utility at baseline** | **HUI3 attribute level change from baseline to week 48** | | | | |
| --- | --- | --- | --- | --- | --- | --- |
| -0.62 | 1.00 | Ambulation: 3 | Emotion: 2 | Pain: 1 | Cognition: 1 |  |
| -0.62 | 1.00 | Ambulation: 5 | Emotion: 1 |  |  |  |
| -0.49 | 0.81 | Pain: 3 | Ambulation: 2 |  |  |  |
| -0.42 | 0.81 | Ambulation: 3 | Cognition: 2 |  |  |  |
| -0.35 | 0.89 | Cognition: 2 | Pain: 1 | Dexterity: 1 | Ambulation: 1 | Speech: 1 |
| -0.33 | 0.88 | Vision: 3 | Pain: 1 | Dexterity: 1 | Ambulation: 1 | Emotion: -1 |
| -0.27 | 0.89 | Speech: 2 | Dexterity: 1 | Ambulation: 1 |  |  |
| -0.27 | 0.95 | Emotion: 2 | Pain: 1 |  |  |  |
| -0.23 | 0.59 | Pain: 2 | Emotion: 1 |  |  |  |
| -0.22 | 0.95 | Cognition: 2 | Dexterity: 2 |  |  |  |
| -0.19 | 0.84 | Cognition: 2 | Ambulation: 1 |  |  |  |
| -0.17 | 0.66 | Emotion: 1 | Ambulation: 1 | Speech: 1 |  |  |
| -0.14 | 1.00 | Pain: 2 |  |  |  |  |
| -0.12 | 1.00 | Pain: 1 | Emotion: 1 |  |  |  |
| -0.12 | 0.97 | Pain: 1 | Hearing: 1 |  |  |  |
| -0.11 | 1.00 | Cognition: 1 |  |  |  |  |
| -0.10 | 1.00 | Ambulation: 1 |  |  |  |  |
| -0.09 | 0.88 | Ambulation: 1 |  |  |  |  |
| -0.07 | 0.79 | Pain: 1 |  |  |  |  |
| -0.07 | 1.00 | Emotion: 1 |  |  |  |  |
| -0.07 | 0.95 | Emotion: 1 |  |  |  |  |
| -0.06 | 0.75 | Emotion: 1 |  |  |  |  |
| -0.05 | 0.87 | Cognition: 1 | Pain: -1 |  |  |  |
| -0.05 | 0.90 | Pain: 1 |  |  |  |  |
| -0.03 | 0.46 | Ambulation: 4 | Dexterity: -4 |  |  |  |
| 0.00 | 1.00 |  |  |  |  |  |
| 0.00 | 0.95 |  |  |  |  |  |
| 0.00 | 1.00 |  |  |  |  |  |
| 0.00 | 0.95 |  |  |  |  |  |
| 0.00 | 0.78 |  |  |  |  |  |
| 0.00 | 0.85 |  |  |  |  |  |
| 0.00 | 1.00 |  |  |  |  |  |
| 0.00 | 0.63 |  |  |  |  |  |
| 0.00 | 0.79 |  |  |  |  |  |
| 0.00 | 0.90 |  |  |  |  |  |
| 0.00 | 0.85 |  |  |  |  |  |
| 0.00 | 1.00 |  |  |  |  |  |
| 0.00 | 0.78 |  |  |  |  |  |
| 0.00 | 0.56 |  |  |  |  |  |
| 0.01 | 0.27 | Ambulation: 2 | Emotion: 1 | Cognition: -1 | Dexterity: -3 |  |
| 0.02 | 0.68 | Speech: 1 | Cognition: -1 |  |  |  |
| 0.04 | 0.84 | Emotion: 1 | Cognition: -1 |  |  |  |
| 0.05 | 0.85 | Pain: -1 |  |  |  |  |
| 0.05 | 0.95 | Pain: -1 |  |  |  |  |
| 0.05 | 0.95 | Pain: -1 |  |  |  |  |
| 0.05 | 0.95 | Pain: -1 |  |  |  |  |
| 0.06 | 0.48 | Cognition: 2 | Emotion: 1 | Pain: -1 | Ambulation: -1 |  |
| 0.06 | 0.52 | Pain: -1 |  |  |  |  |
| 0.10 | 0.90 | Ambulation: -1 |  |  |  |  |
| 0.11 | 0.68 | Dexterity: 1 | Pain: -1 | Cognition: -1 |  |  |
| 0.12 | 0.88 | Pain: -1 | Emotion: -1 |  |  |  |
| 0.12 | 0.43 | Emotion: 2 | Speech: -1 | Ambulation: -2 |  |  |
| 0.15 | 0.64 | Emotion: 1 | Cognition: -3 |  |  |  |
| 0.15 | 0.85 | Pain: -1 | Ambulation: -1 |  |  |  |
| 0.19 | 0.81 | Ambulation: -2 |  |  |  |  |
| 0.28 | 0.08 | Ambulation: 4 | Speech: -1 | Pain: -2 | Cognition: -4 |  |
| 0.34 | 0.66 | Emotion: -1 | Dexterity: -1 | Ambulation: -1 | Pain: -2 |  |
