# Supplementary material for: Factors associated with the health-related quality of life among people with Duchenne muscular dystrophy: a study using the Health Utilities Index (HUI)
Source: Health Qual Life Outcomes. 2022 Jun 11;20:93. doi: 10.1186/s12955-022-02001-0 (PMC9188127; doi:10.1186/s12955-022-02001-0)
Supplement: Supplementary file 4 — Additional file 4: Appendix Table 4. Observed changes in HUI2 utility and attribute levels from baseline to week 48, with each row representing an individual patient. [file 12955_2022_2001_MOESM4_ESM.docx]

Appendix table 4: Observed changes in HUI2 utility and attribute levels from baseline to week 48, with each row representing an individual patient.

| **HUI2 utility change from baseline to week 48** | **HUI2 utility at baseline** | **HUI2 attribute level change from baseline to week 48** | | | | |
| --- | --- | --- | --- | --- | --- | --- |
| -0.49 | 0.83 | Pain: 3 | Selfcare: 3 | Mobility: 1 |  |  |
| -0.40 | 0.97 | Emotion: 3 | Pain: 1 |  |  |  |
| -0.36 | 0.80 | Mobility: 3 | Selfcare: 3 |  |  |  |
| -0.32 | 0.80 | Selfcare: 2 | Cognition: 1 | Emotion: 1 | Mobility: 1 |  |
| -0.24 | 0.93 | Emotion: 2 |  |  |  |  |
| -0.22 | 0.97 | Emotion: 2 | Pain: 1 |  |  |  |
| -0.22 | 0.97 | Emotion: 2 | Selfcare: 1 |  |  |  |
| -0.22 | 0.92 | Sensation: 2 | Emotion: 1 | Mobility: 1 |  |  |
| -0.20 | 0.78 | Selfcare: 2 | Emotion: 1 |  |  |  |
| -0.15 | 0.87 | Sensation: 2 | Mobility: 1 |  |  |  |
| -0.12 | 0.88 | Sensation: 2 | Mobility: 1 | Pain: 1 | Emotion: -1 |  |
| -0.12 | 0.72 | Emotion: 1 | Mobility: 1 |  |  |  |
| -0.10 | 1.00 | Emotion: 1 | Pain: 1 |  |  |  |
| -0.10 | 0.97 | Emotion: 1 | Selfcare: 1 |  |  |  |
| -0.09 | 0.77 | Mobility: 2 | Cognition: 1 | Selfcare: 1 | Emotion: -1 |  |
| -0.09 | 0.68 | Pain: 1 |  |  |  |  |
| -0.08 | 0.79 | Sensation: 2 | Cognition: -1 |  |  |  |
| -0.07 | 0.85 | Cognition: 1 | Selfcare: 1 |  |  |  |
| -0.07 | 0.94 | Emotion: 1 |  |  |  |  |
| -0.07 | 0.92 | Emotion: 1 | Mobility: 1 | Selfcare: -1 |  |  |
| -0.07 | 0.91 | Emotion: 1 |  |  |  |  |
| -0.06 | 1.00 | Mobility: 1 | Selfcare: 1 |  |  |  |
| -0.05 | 1.00 | Cognition: 1 |  |  |  |  |
| -0.05 | 0.55 | Mobility: 1 | Cognition: -1 |  |  |  |
| -0.05 | 0.97 | Cognition: 1 |  |  |  |  |
| -0.04 | 0.74 | Cognition: 1 |  |  |  |  |
| -0.03 | 1.00 | Pain: 1 |  |  |  |  |
| -0.03 | 1.00 | Pain: 1 |  |  |  |  |
| -0.03 | 1.00 | Pain: 1 |  |  |  |  |
| 0.00 | 0.95 |  |  |  |  |  |
| 0.00 | 0.82 |  |  |  |  |  |
| 0.00 | 0.97 |  |  |  |  |  |
| 0.00 | 1.00 |  |  |  |  |  |
| 0.00 | 1.00 |  |  |  |  |  |
| 0.00 | 1.00 |  |  |  |  |  |
| 0.00 | 0.94 |  |  |  |  |  |
| 0.00 | 0.74 |  |  |  |  |  |
| 0.00 | 1.00 |  |  |  |  |  |
| 0.00 | 1.00 |  |  |  |  |  |
| 0.00 | 0.93 |  |  |  |  |  |
| 0.03 | 0.97 | Selfcare: -1 |  |  |  |  |
| 0.05 | 0.48 | Mobility: 2 | Selfcare: 2 | Pain: -1 | Cognition: -2 | Sensation: -2 |
| 0.06 | 0.94 | Mobility: -1 | Selfcare: -1 |  |  |  |
| 0.06 | 0.94 | Mobility: -1 | Pain: -1 |  |  |  |
| 0.07 | 0.84 | Emotion: -1 |  |  |  |  |
| 0.07 | 0.65 | Pain: 1 | Selfcare: -1 |  |  |  |
| 0.07 | 0.93 | Emotion: -1 |  |  |  |  |
| 0.11 | 0.79 | Cognition: -1 | Emotion: -1 |  |  |  |
| 0.13 | 0.54 | Pain: -1 | Selfcare: -1 |  |  |  |
| 0.13 | 0.87 | Emotion: -1 | Mobility: -1 | Pain: -1 |  |  |
| 0.15 | 0.53 | Emotion: 1 | Mobility: -1 | Sensation: -2 |  |  |
| 0.17 | 0.74 | Selfcare: -2 |  |  |  |  |
| 0.17 | 0.79 | Cognition: -1 | Emotion: -1 | Pain: -1 | Selfcare: -1 |  |
| 0.26 | 0.74 | Emotion: -1 | Pain: -1 | Mobility: -2 |  |  |
