# Supplementary material for: Factors associated with the health-related quality of life among people with Duchenne muscular dystrophy: a study using the Health Utilities Index (HUI)
Source: Health Qual Life Outcomes. 2022 Jun 11;20:93. doi: 10.1186/s12955-022-02001-0 (PMC9188127; doi:10.1186/s12955-022-02001-0)

Appendix figure 1 Changes in HUI attribute levels from baseline to week 48 among patients with (A) a decline in HUI utility of at least 0.1, (B) an improvement in HUI utility of at least 0.1, (C) a decline in HUI utility of at least 0.03, and (D) an improvement in HUI utility of at least 0.03, between baseline and week 48

A. Patients with a decline in HUI utility of at least 0.1

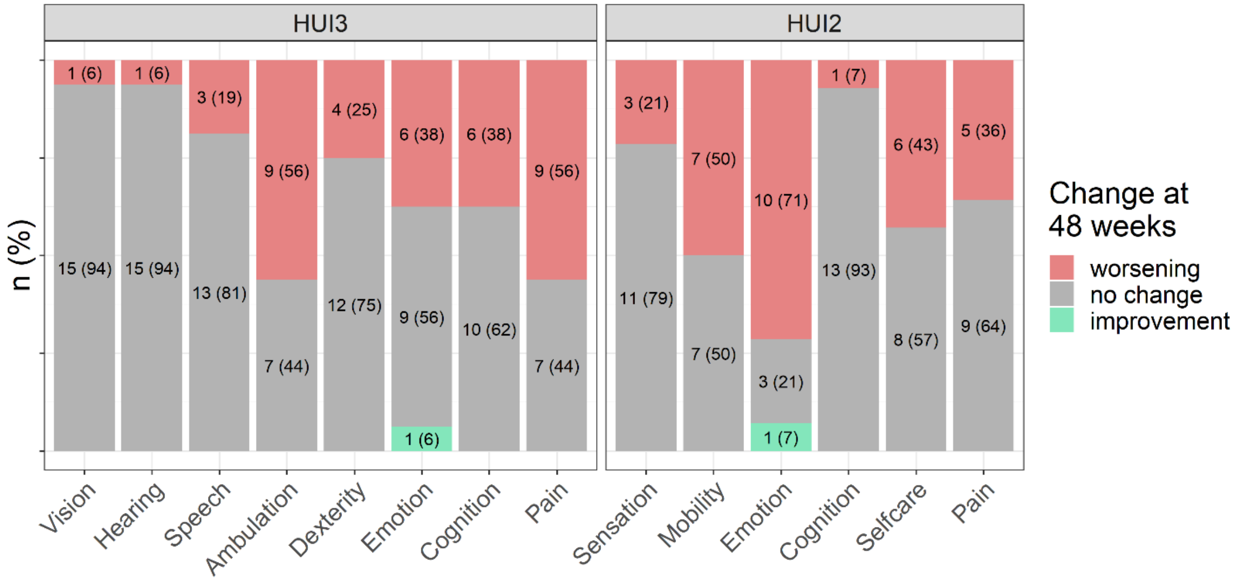

B. Patients with an improvement in HUI utility of at least 0.1

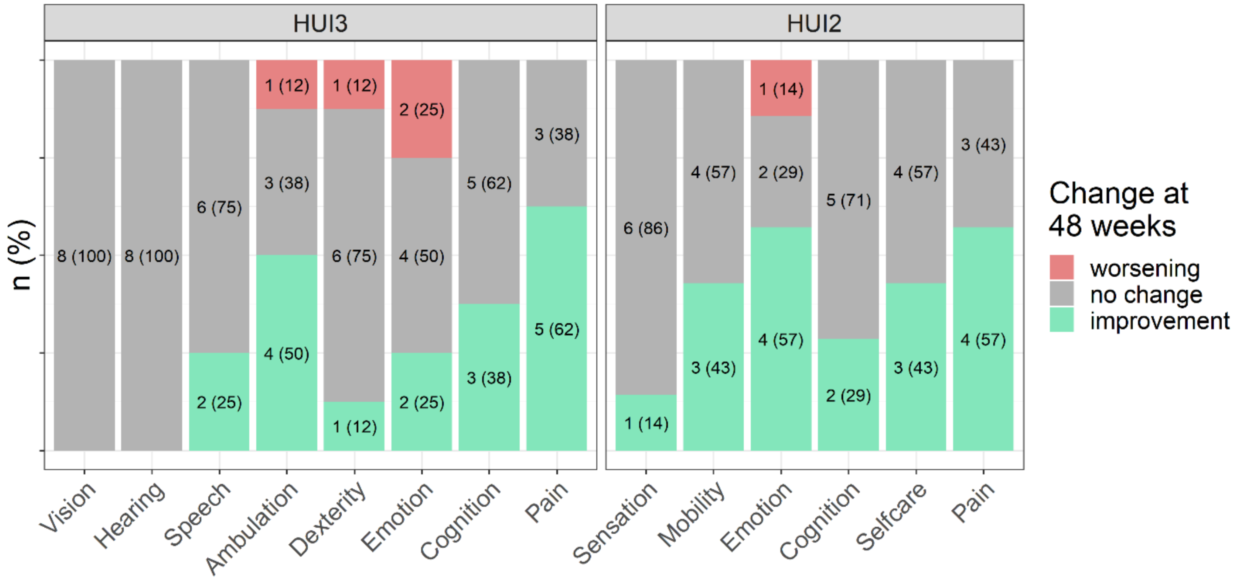

C. Patients with a decline in HUI utility of at least 0.03

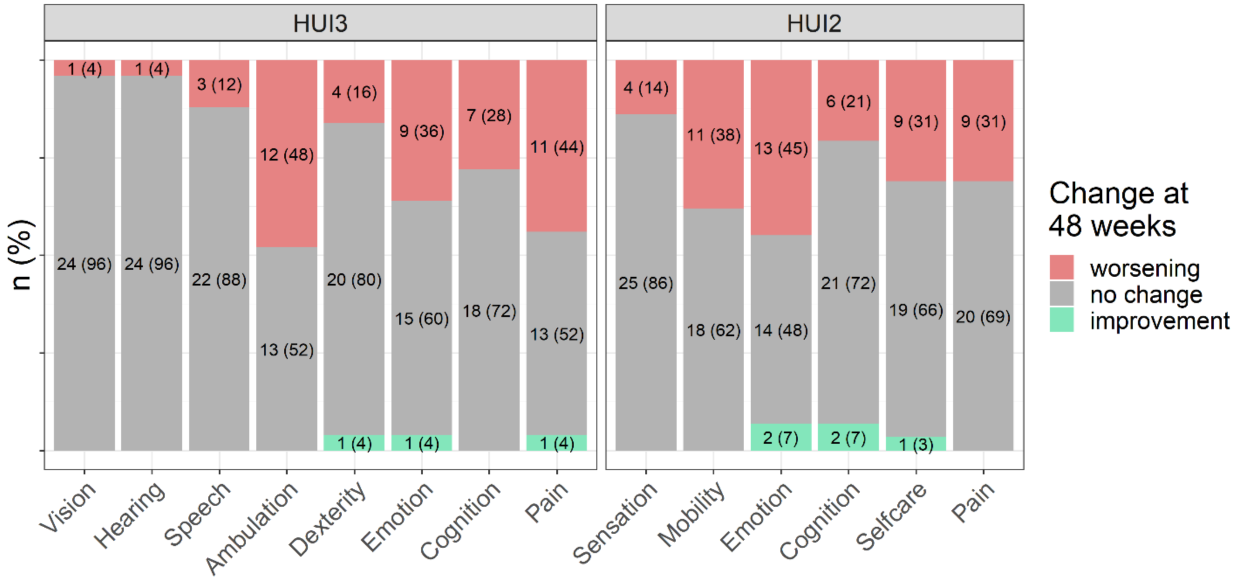

D. Patients with an improvement in HUI utility of at least 0.03

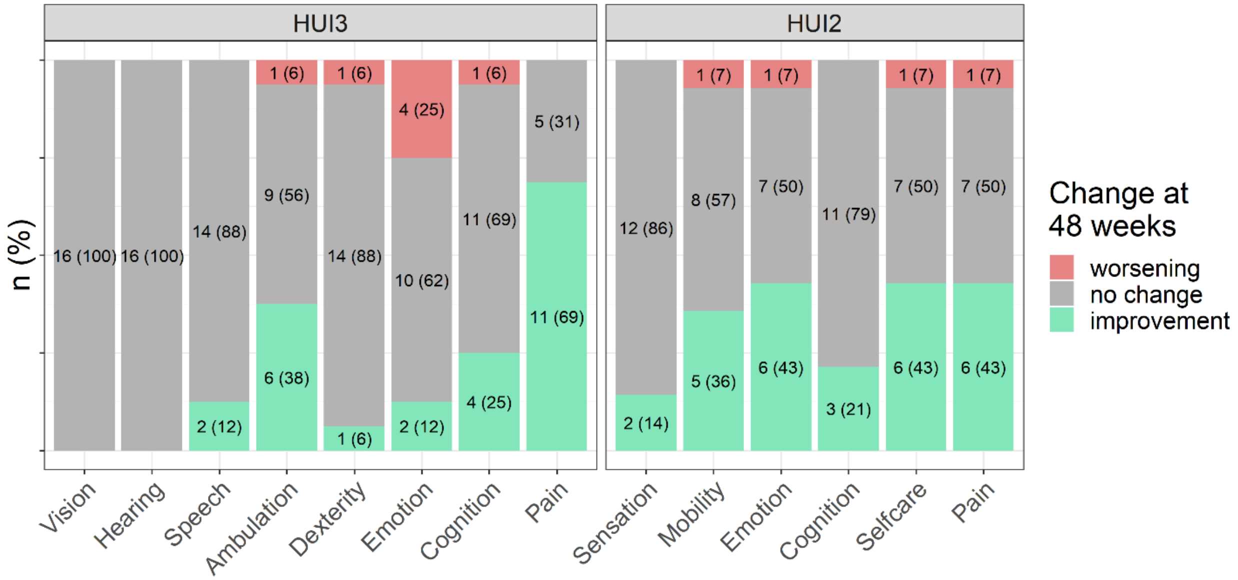

Supplement: Supplementary file 5 — Additional file 5: Appendix Figure 1. Changes in HUI attribute levels from baseline to week 48 among patients with (A) a decline in HUI utility of at least 0.1, (B) an improvement in HUI utility of at least 0.1, (C) a decline in HUI utility of at least 0.03, and (D) an improvement in HUI utility of at least 0.03, between baseline and week 48. [file 12955_2022_2001_MOESM5_ESM.pdf]
