# Supplementary material for: Factors associated with the health-related quality of life among people with Duchenne muscular dystrophy: a study using the Health Utilities Index (HUI)
Source: Health Qual Life Outcomes. 2022 Jun 11;20:93. doi: 10.1186/s12955-022-02001-0 (PMC9188127; doi:10.1186/s12955-022-02001-0)

Appendix figure 2 HUI3 and HUI2 utility by time since LOA, with a black smoothing line and colors denoting individual patients (n=6)

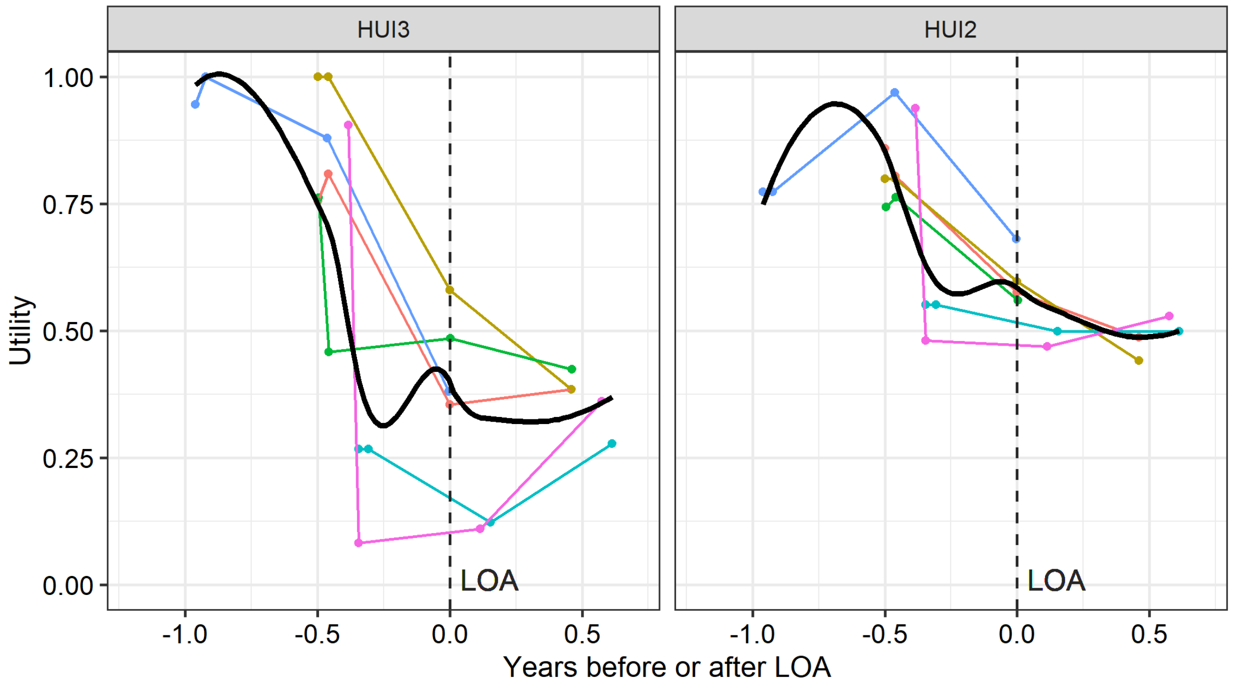

Supplement: Supplementary file 6 — Additional file 6. Appendix Figure 2. HUI3 and HUI2 utility by time since LOA, with a black smoothing line and colors denoting individual patients (n=6). [file 12955_2022_2001_MOESM6_ESM.pdf]
